# Projective in Time: A Systematic Review on the Use of Construction Projective Techniques in the Digital Era—Beyond Inkblots

**DOI:** 10.3390/children12040406

**Published:** 2025-03-24

**Authors:** Giada Santillo, Rita Chiara Morra, Dario Esposito, Maria Romani

**Affiliations:** Department of Human Neuroscience, Sapienza University of Rome, 00185 Rome, Italy; giada.santillo@uniroma1.it (G.S.); ritachiara.morra@uniroma1.it (R.C.M.); maria.romani@uniroma1.it (M.R.)

**Keywords:** projective techniques, projective tests, drawing techniques, children, adolescents, children apperception test, thematic apperception test, family drawing test, house-tree-person test, draw a person test

## Abstract

Background: Projective instruments have historically been used to explore unconscious dynamics and personality traits in children and adolescents. However, concerns about their psychometric properties have led to ongoing debates in clinical psychology. This review aims to critically reassess the use of construction projective techniques in clinical settings for individuals aged 4 to 18 years, also examining the available psychometric data reported in recent scientific literature, excluding the Rorschach Test due to its extensive coverage in existing literature. Methods: A systematic review was conducted following PRISMA guidelines. PubMed, Psychoanalytic Electronic Publishing (PEP), and the Cochrane Library were searched for studies published between 2010 and 2024. Inclusion criteria encompassed studies focusing on construction projective techniques administered to clinical and non-clinical samples aged 4 to 18 years. Results: From an initial pool of 641 articles, 25 met the inclusion criteria. These studies indicate that construction projective techniques remain valuable in accessing children’s and adolescents’ inner worlds, particularly in diagnostic and therapeutic contexts. However, 13 of the 25 analyzed studies lack detailed psychometric evaluations, and the overall methodological quality was medium-low (4.4/9). Recent adaptations of these techniques show promise in increasing their applicability and relevance. Conclusions: Constructive projective tests continue to offer unique insights into the psychological functioning of young individuals. While not definitive diagnostic tools, they serve as effective supplements in clinical assessments and therapeutic interventions when applied with awareness of their limitations.

## 1. Introduction

In psychology, the term ’projective test’ refers to the use of ambiguous, unstructured stimuli designed to elicit responses in which individuals ‘project’ aspects of themselves, assigning personal meaning based on their life experiences [1]. Projective techniques may be defined as methods that use ambiguous stimuli to elicit responses that reveal aspects of an individual’s personality, thoughts, and emotions, often bypassing conscious defenses and providing insights into unconscious processes [2,3,4,5]. These techniques have been found to be useful for understanding children’s attitudes, fears, needs, wishes, maturity level, stressors and self-perception in relation to their family and significant others and for assessing children’s personality and psychological disorders. These techniques provide a convenient way to explore the internal worlds of children, serving both as clinical diagnostic tools and as research instruments [6]. These techniques offer insights into emotional perceptions, functional levels, and environmental interactions [7]. Moreover, they often highlight resilience factors essential for positive developmental trajectories, revealing individual strengths and interests. The key advantages of projective tests lie in their simplicity, cost-effectiveness, sensitivity, and cultural adaptability, making them practical for evaluating emotional well-being. Their characteristics include: (a) ambiguity of the stimulus: individuals are required to organize and interpret the stimuli, thus allowing the content and structure of their personality to emerge; (b) variety of responses: responses are open-ended, without criteria for correctness or falsity, and are not judged as right or wrong; (c) interpretative analysis: examiners analyze responses and provide interpretations based on observed elements.

Projective techniques can be categorized into five broad and sometimes overlapping groups: construction, association, completion, selection (or arrangement), and expression techniques [1,8] (Table 1).

The aim of this study is to conduct a scoping review of the recent literature (2010–2024) on the projective tests most frequently employed in developmental clinical practice—specifically, those that can be categorized as construction projective techniques according to our classification (see Table 1). By integrating drawing and storytelling, these construction projective techniques facilitate indirect access to significant emotional and conflictual themes, thereby enabling the exploration of complex psychological dynamics that may remain inaccessible through clinical interviews or standardized assessments. Historically, instruments such as the Rorschach, Thematic Apperception Test (TAT), Human Figure Drawing, and their derivatives have been widely used in clinical settings with children and adolescents [1,18,19]. However, given the extensive recent reviews dedicated exclusively to the Rorschach [20,21], this review deliberately excludes it to avoid redundancy. Moreover, this review examines recent adaptations, updates, and revisions of constructive projective tests, providing insights into their current applicability and evolving role in clinical practice.

## 2. Materials and Methods

**Figure 1:**
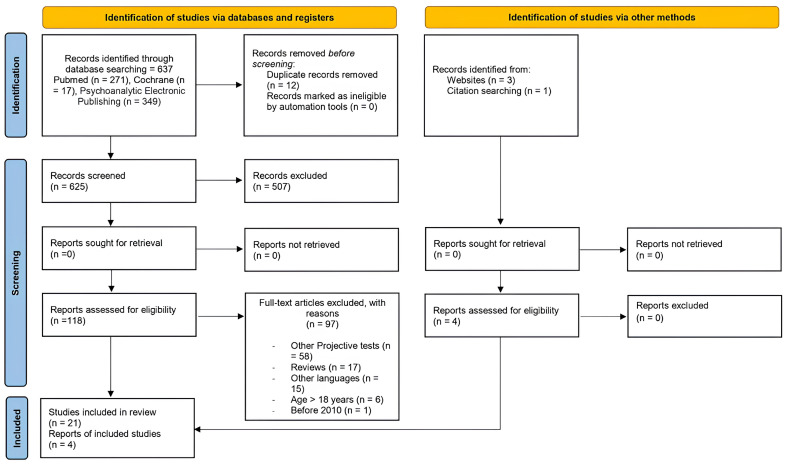
PRISMA 2020 flow diagram for new systematic reviews which included searches of databases, registers and other sources [22].

The present review followed PRISMA guidelines for systematic scoping reviews [22]. Figure 1 depicts the PRISMA flow diagram, describing the selection process in detail. We searched PubMed Central database, PEP—Psychoanalytic Electronic Publishing, and Cochrane Library using the search terms specified subsequently, for publications published from January 2010 to August 2024. As this is a scoping review—focused on mapping the literature rather than addressing a specific health-related outcome—it was not eligible for PROSPERO registration. Study inclusion criteria were: (1) studies that focused on constructive projective techniques as defined above; (2) empirical studies, including cross-sectional, prospective studies, and case reports; (3) participants aged 4 to 18 years. Studies with mixed-age samples were included if the data on the 4–18 age subgroup were reported separately; (4) articles discussing any aspect of the application, adaptation, or revision of these techniques; (5) publications in English. Exclusion criteria: (1) studies exclusively addressing the Rorschach test; (2) review articles (although their reference lists were examined to identify additional eligible studies).

The following search terms were used: (projective techniques OR projective test OR drawing techniques OR thematic apperception test OR children apperception test OR family drawing test OR house-tree-person OR draw-a-man OR draw-a-person) AND (children OR child OR adolescents OR adolescent). A comprehensive search was conducted using the following strategy: additional relevant studies were identified by scanning reference lists of trials identified in the initial searches. Titles and abstracts of references identified by the electronic search strategies described above were selected according to their relevance. Then the full texts of all remaining articles were evaluated and included with the same criteria of relevance. Quality assessment of the retrieved publications was performed employing a quality index derived and adapted from the Newcastle-Ottawa Scale (NOS), a widely used tool based on a 9-star model, where higher scores indicate better quality and lower risk of bias [23]. For further quality evaluation details, see Supplementary Tables S1 and S2.

## 3. Results

From the initial database search, 641 potentially eligible articles were retrieved; subsequently, duplicates were removed, 629 manuscripts were then screened by reading title and abstract, and unfocused articles were removed. 122 manuscripts were then screened by reading the full text. Inclusion and exclusion criteria were then applied for further screening of the articles: of these, 25 fulfilled all the predefined criteria and were included in the present systematic review.

### 3.1. General Characteristics of the Studies

All included studies were published between 2010 and 2024. The quality index of the retrieved studies ranged between 2 and 8 points out of 9, with an average of 4.36 and a median score of 4, which corresponds to “fair quality” according to the Agency for Healthcare Research and Quality (AHRQ) standards [24]. Thirteen studies used a cross-sectional design, five studies used a case-control design, seven studies applied other methods (two case-reports, two longitudinal studies, two qualitative studies and one retrospective study). Five of the studies included control groups. Sample sizes of the studied groups varied between 1 and 1757, with a minimum age of 4 and a maximum of 18 years. Globally, a total of 3.854 participants were evaluated. Notably, 13 out of the 25 studies did not report any psychometric data on the projective tests used, nor did they cite prior research to support their validity and reliability, raising concerns about the methodological robustness of these studies.

The following construction projective techniques were identified and analyzed based on the systematic search conducted for this review (Table 2):

Table 3 provides a detailed description of the included studies.

### 3.2. Main Focus of Included Studies

Projective construction techniques were taken into consideration in all 25 of the examined studies. 9 of these studies concentrate on the use of a single narrative projective technique, which uses ambiguous stimuli to construct stories in order to gain insight into children’s and teenagers’ inner lives. Of these 9 articles, 2 examine the CAT [69,70], 2 examine the Bears Family Projective Test [42,66], and 5 examine the use of the TAT [59,67,68,72,73]. 13 studies, however, rely on the sketching of specific figures or images and employ a single graphic projective style. Of these, 7 studies analysed the DAP/DAM technique [60,61,62,63,64,65,74], 2 analysed the DAF technique [54,55], 2 the HTP technique [52,58], 1 method known as “Drawn a Story Technique” [53] and 1 the method known as BND [75]. Additionally, some studies take into account multiple projective approaches. For example, one study looks at the value of both CAT and DAF [71], another at the use of DAP and DAF [56], and a third at DAF and HTP [57]. The Rorschach Test, which is the primary association projective technique and will not be discussed in this paper, is included in two of the reviewed publications [59,73] in addition to analyzing projective construction approaches. Since every study focuses on developmental age, only samples, both male and female, between the ages of 4 and 18 are looked at. A larger age range is considered only in one study (25 people between the ages of 10 and 41, 13 of whom were patients between the ages of 12 and 18).

### 3.3. Diagnosis of Samples Included in Studies

Twelve of the studies reviewed considered clinical samples (patients with a neuropsychiatric diagnosis, diagnosed with organic diseases or followed in hospital for pathogenic conditions), six focus on non-clinical samples and seven deal with both clinical and non-clinical samples (the five case-control studies and three studies analyzing one clinical and one non-clinical sample, but these were not compared to assess a specific condition).

The clinical samples presented several neuropsychiatric diagnoses: autism spectrum disorder (ASD) [58,65,66], attention deficit hyperactivity disorder (ADHD) [56,70], depressive symptoms [52], anxiety symptoms [60], schizophrenia spectrum, schizotypal disorder [75], emotional and relational difficulties [42], behavioral disorders associated with learning disabilities [61], emotional problems secondary to abuse [63], attachment disorders [71], post-traumatic stress disorder (PTSD) [59], non-suicidal self-injury (NSSI) [69,72], attempted suicide [73], epilepsy [60], preterm birth [62], cognitive impairment related to genetic conditions [57]. In another report [74], the case of a girl diagnosed with acute lymphoblastic leukaemia was analyzed.

## 4. Discussion

The reviewed studies indicate that Constructive Projective Tests may provide valuable insights into individuals’ psychic functioning, emotional lives, and relational worlds. However, many of these historically established instruments have not been significantly re-evaluated for their psychometric properties since 2010. Despite this limitation, they are widely used due to their cost-effectiveness and non-invasive nature. For example, these tests have been applied in diverse clinical contexts, including studies with refugees [54], populations at risk of abuse or maltreatment [63], children with rare genetic conditions [57], autism [65,66], epilepsy [60], very preterm infants [61,62], children with leukemia [74], individuals with non-suicidal self-injury behaviors [69,72], and adolescents following suicide attempts [73]. Nevertheless, nearly half of the examined studies did not provide an in-depth examination of the psychometric properties of the projective tests employed, which remains a major limitation in their empirical applicability. An outline of the individual tests and their current use, as indicated by the available evidence, is provided below.

### 4.1. Thematic Apperception Test

Several included studies [59,67,68,72,73] underscored the enduring versatility and significance of the TAT as a psychological investigative tool across diverse contexts and populations. The TAT remains a valuable instrument for examining complex psychological constructs, including emotional dynamics, attachment styles, executive functions, object relations, and trauma. The analysis of narratives elicited through the TAT provides access to implicit and unconscious content, reinforcing its utility in psychological assessment and a comprehensive understanding of individuals.

The TAT has been proposed [59] in conjunction with other projective and non-projective assessment tools to improve the identification and understanding of bullying, thereby contributing to more effective intervention strategies. This study employed the TAT, alongside additional projective instruments and interviews, to examine unconscious psychological processes and to identify vulnerabilities, traumatic experiences, psychological resources, and behavioral patterns among bullied adolescents. The findings suggest that the TAT facilitates the identification of anxieties linked to bullying-related traumatic experiences through the content of the elicited narratives. Ultimately, the study advocates for a mixed-methods approach, incorporating projective tests, to enhance understanding of bullying and its psychological ramifications. The research findings may contribute to refining the criteria for identifying bullying, improving assessment protocols, and developing new strategies for prevention and intervention for affected individuals.

Article [67] explores the implementation of a novel assessment protocol, the Adolescent Attachment Protocol (AAP), which is grounded in the TAT and aims to evaluate attachment styles in adolescents. The AAP distinguishes between secure and insecure attachment patterns by qualitatively analyzing of narrative themes. The study highlights that the TAT, via the qualitative examination of generated narratives, provides profound insights into adolescents’ mental representations of attachment, surpassing the depth offered by self-report measures. By employing the TAT as a projective assessment tool, the AAP proves to be a promising methodological approach for evaluating attachment styles in adolescence, offering a qualitative perspective on the cognitive and emotional representations that shape interpersonal relationships.

In article [68], the TAT is used to assess executive functions (EF) and social competence (SC) in kindergarten-aged children. The findings indicate that this projective instrument serves as a valid predictor of social competence in real-life contexts, underscoring its applicability in early childhood psychological assessment.

Article [72] examines the narrative representations elicited by the TAT among adolescents who engage in non-suicidal self-injury (NSSI). The study reveals that adolescents with NSSI tendencies exhibit greater complexity in their narrative depictions of individuals compared to their non-NSSI peers. Furthermore, these adolescents show less commitment to moral values and ethical standards. These findings suggest that the TAT may serve as an effective tool for assessing the internal representations of interpersonal relationships in individuals exhibiting self-injurious behaviors.

Finally, article [73] demonstrates that projective techniques, including the TAT, contribute to a deeper understanding of the psychological mechanisms underlying suicidal behaviors in adolescents. By providing access to the latent motivations driving such actions, projective tests facilitate therapeutic interventions aimed at addressing the pathological dimensions associated with suicidality. As such, these techniques serve as critical instruments in both psychological assessment and psychotherapy, providing a way to explore and mitigate underlying distress in at-risk populations.

### 4.2. Children’s Apperception Test

Studies 53–55 reaffirm the enduring effectiveness of projective techniques such as the CAT, often adapted and updated to align with contemporary contexts, in diagnostic settings aimed at exploring the inner world of children and adolescents. These studies further suggest that the CAT can serve not only as a diagnostic instrument but also as a therapeutic mediator. Moreover, the test is used alongside other projective techniques, such as drawing-based assessments, to enhance its efficacy in psychological evaluation.

In article [69], a modified version of the CAT is introduced, in which human figures are used as stimuli, requiring the child to narrate and describe a story around them. This variant, known as the Child Apperception Test with Human Figures (CAT-H), bears similarities to Winnicott’s squiggle game [76]. The study demonstrates that this instrument facilitates the expression of internal conflicts, particularly the tension between submissiveness and the desire for autonomy, as well as feelings of vulnerability and helplessness. This enhanced expressive capacity enables a deeper understanding of adolescent distress. Beyond its diagnostic application, this variant of the CAT also functions as a therapeutic mediator, allowing adolescents to reflect on their emotional states. Another interesting point is that this study proves CAT may be particularly valuable in adolescence, as it provides a framework for revisiting early childhood experiences. This process aligns with Winnicott’s (1968–1999) [76] conceptualization of the “reappearance of the same problems that were present in early life,” facilitating adolescents’ re-elaboration of early identifications that contribute to the formation of their sense of self.

Another adaptation of the classical CAT, the Children’s Apperception Test—Animal Form (CAT-A), is examined in article [70]. In this version, animal figures replace human characters. This modification has proven particularly effective with children, both as a productive tool for facilitating dialogue—underscoring its role as a communication bridge between the examiner and the child—and as a means of allowing children to articulate their internal experiences and emotional struggles through the narratives they construct in response to the test stimuli.

Finally, article [71] investigates two projective construction techniques: the CAT and the DAF. These tests are employed to assess whether the quality of children’s attachment relationships is associated with their capacity for mentalization and their artistic expressions. The study reveals that the CAT and DAF might serve as instruments in exploring how children’s early relational experiences and their mentalization development manifest through language (as assessed by the CAT) and artistic production (as examined through the DAF). The findings indicate that these tools are instrumental in understanding children’s internal worlds, their representations of interpersonal relationships, and their ability to understand and interpret both their own and others’ mental states.

### 4.3. Draw a Man/Draw a Person

Several studies [56,60,61,62,63,64,65,74] highlighted the versatility of the DAP test and its variants across diverse diagnostic and research contexts, demonstrating its utility in assessing children’s internal representations, emotional states, and cognitive development. These studies indicate that these projective techniques can be employed to evaluate a broad spectrum of conditions, including anxiety disorders, cognitive disabilities, behavioral difficulties, childhood sexual abuse, hyperadaptation, and other emotional challenges, as well as to compare different groups of children. Findings from these projective assessments can be integrated with other diagnostic measures to provide valuable insights for therapeutic interventions and support strategies.

In study [56], two projective techniques—DAP and DAF—were used to compare the drawing performance of children diagnosed with Attention Deficit Hyperactivity Disorder (ADHD) and those with typical development. The study aimed to assess impulsivity and emotional difficulties through graphical representation. Significant differences were observed in drawing performance between the two groups, suggesting that projective tests may still be valuable in identifying difficulties in children with ADHD, although they should be supplemented with additional assessments. Furthermore, the authors propose that the DAP and DAF, due to their ease of administration and applicability, could serve as preliminary screening tools for educators to identify children at risk of ADHD and refer them to specialists for further evaluation and appropriate intervention.

In study [60], the authors propose the use of the DAP as a projective tool to assess anxiety in pediatric patients with neuropsychiatric disorders, such as epilepsy. The drawings provided complementary data to questionnaire-based measures, offering a more comprehensive understanding of participants’ emotional experiences. The study concludes that the DAP serves as a useful starting point for investigating anxiety symptoms in this population, opening avenues for further assessment and discussion. Additionally, the research suggests that the DAP method could aid in identifying children at risk of developing adjustment disorders related to their medical conditions.

In study [61], the classical DAM test, originally developed by Goodenough, was employed to investigate correlations between DAM scores and cognitive and behavioral disabilities in children born preterm. The study found a significant association between lower DAM scores and cognitive and behavioral impairments; however, the test was not deemed sufficiently reliable as a standalone screening or diagnostic tool. Despite these limitations, the authors suggest that human figure drawings offer valuable insights into development for children born very prematurely. They recommend further research with larger sample sizes and more sophisticated analytical methods to improve the reliability of the DAM or to develop new assessment tools based on human figure drawing.

Study [62] used the DAP to evaluate developmental differences between children born preterm and those born at term, specifically in relation to cognitive and motor development. Findings revealed that preterm children exhibited lower scores on the DAP compared to their full-term counterparts, suggesting a possible delay in cognitive and motor skills. The study concludes that the DAP may serve as an early indicator of cognitive and motor deficits in children born very preterm. A low score on the DAP could serve as an early warning sign, warranting further investigation using standardized cognitive and motor assessments.

In study [63], a variant of the DAP—the Two Human Figures (T2F) test—was used, featuring a specific scoring system. The objective was to determine whether children’s drawings in at-risk or neglectful environments displayed graphical indicators suggestive of childhood sexual abuse. The study revealed that children in high-risk situations exhibited distinctive graphic indicators of sexual abuse, including body distortions, alterations in the depiction of eyes and hands, and elevated scores on the emotional indicators scale of the T2F. Consequently, the DAP, and particularly the T2F variant, emerges as a promising tool for identifying potential maltreatment or experiences of sexual abuse in vulnerable children.

In article [64], the DAM was utilized to assess hyperadaptive tendencies in children by analyzing drawing scores and characteristics. The test was employed to compare the drawing performance of children exhibiting hyperadaptation with that of control children, investigating whether hyperadaptive children displayed distinct graphical features. The findings suggest that the test could potentially be used to identify children at risk of developing psychological difficulties associated with hyperadaptation at an early stage.

Study [65] examined the use of the DAM to compare the drawing performance of children with ASD and those with typical development (TD). Additionally, the study investigated whether the participant of the drawing (self vs. others) influenced performance. The results indicated that children with ASD scored lower on the DAM than TD children; however, no significant differences were observed between self-representations and other depictions. Based on these findings, the authors argue that the use of this test in clinical practice for individuals with ASD may be limited, as it does not provide sufficient items to inform clinical decision-making. Nevertheless, they suggest that this type of projective test may hold potential in basic research by elucidating relationships between drawing performance and neuropsychological characteristics, thereby providing insights into autistic cognition and functioning.

Finally, in article [74], a variant of the DAP—known as the Draw-A-Person: Screening Procedure for Emotional Disturbances (DAP:SPED)—was employed as a screening tool for emotional disturbances in a pediatric oncology patient. The DAP:SPED is a psychometrically validated measure [74] designed for actuarial-based assessment. The test requires the examinee to draw a man, a woman, and themselves on separate pages, with a five-minute limit per drawing. Unlike self-report measures, the DAP:SPED is less susceptible to social desirability bias and can be particularly useful in clinical settings where time and verbal ability may be constraints [74]. In this study, the DAP:SPED indicated a high likelihood of emotional adjustment difficulties, despite the fact that self-report questionnaires did not detect such issues. The qualitative analysis of the drawings provided critical insights into the child’s perceptions that would not have emerged through questionnaires alone. The study underscores the importance of integrating both quantitative and qualitative methodologies to achieve a more comprehensive understanding of patients’ emotional states and psychological well-being.

### 4.4. Draw a Family

Several studies [54,55,57] demonstrate that drawing techniques can be particularly effective in understanding children and adolescents who experience difficulties in verbally expressing their emotions and thoughts, due to language and cultural barriers or medical conditions. According to these studies, the DAF test serves this purpose by revealing not only family dynamics but also the individual’s fears, needs, conflicts, and self-perception.

Some authors [54] focus on the use of drawing as a methodological tool for understanding the family dynamics of immigrant children. These children often struggle to articulate their emotions verbally, whether due to linguistic and cultural barriers or because of difficulties in externalizing their experiences. Drawing, therefore, offers a non-verbal alternative for expressing their emotions and thoughts. The analysis of these children’s drawings enabled researchers to assess their perceptions of their environment, relationships with parents and siblings, and overall family dynamics. Signs of family conflict may manifest in the drawings through distortions in the depiction of family members or the omission of family members. According to the authors, the use of the family drawing test contributes to a deeper understanding of the needs of immigrant families and can inform the development of effective interventions aimed at promoting their well-being.

An exploratory study presents a newly developed projective test tailored to contemporary children [55]: the Draw-a-Family Picture Test (DAFPT). This test was designed based on the principles of existing projective techniques, incorporating elements from well-established assessments such as the Draw-a-Person Test, the Bender-Gestalt Test, and the Goodenough Intelligence Test [30]. Expert evaluations, as well as feedback from parents, suggest that the DAFPT is a highly suitable instrument for identifying children’s attitudes toward family members, their fears, needs, level of maturity, stressors, gender identity tendencies, and self-perception. The study highlights the potential of this updated tool in fostering a better understanding of children’s emotional states and facilitating their adaptation both at home and in school settings.

Finally, study [57] evaluates the applicability of projective techniques such as the HTP test and the DAF test in individuals diagnosed with Prader-Willi syndrome (PWS). A significant challenge for individuals with PWS is their difficulty in identifying and verbalizing emotions, which frequently manifests through problematic behaviors. Both tests provided valuable insights into self-image, environmental perceptions, conflicts, fears, and anxieties. The drawing of the house was found to reflect emotions experienced within social contexts, the depiction of the tree revealed unconscious aspects of personality, and the human figure drawing illustrated body image perception. The family drawing test, in particular, facilitated the exploration of family dynamics, the individual’s role within the family unit, and emotional identifications. These projective techniques thus offer a means of investigating conflicts, emotions, and thoughts that individuals with PWS may be unable to articulate verbally. The study underscores the utility of projective assessments in gaining insight into individuals with intellectual disabilities, as these methods provide critical insights when direct verbal communication is limited or insufficient.

Despite the aforementioned positive results, there is ongoing debate regarding the appropriateness of utilizing this test during adolescence [77]. Some scholars argue that because adolescents are capable of achieving visual realism in their drawings, the clinical value of their illustrations is limited. Others, however [77,78], contend that adolescents’ drawings provide access to unconscious content and facilitate the articulation of emotions. Additionally, as adolescents often exhibit reluctance toward participating in face-to-face interviews or surveys, drawing serves as an alternative means for them to express their emotions and desires related to family relationships.

### 4.5. House-Tree-Person

The HTP test employs a qualitative, subjective analysis in its scoring system to assess personality characteristics. However, similar to other qualitative projective techniques, its validity remains a subject of debate due to the limited empirical evidence supporting its interpretative frameworks and the variability in assessment outcomes across different evaluators.

Studies [52,58] demonstrate that the HTP test, including its variant, the Synthetic House-Tree-Person (S-HTP) test, continues to be a valid projective tool for investigating emotional and developmental aspects in children and adolescents. According to the authors, the HTP test provides valuable insights for screening depressive disorders (through the specific analysis of drawing characteristics) as well as for screening neurodevelopmental disorders (by assessing the ability to complete the test and evaluating mental age).

In article [52], the authors focus on the application of the HTP test to identify drawing characteristics associated with depressive symptoms in middle school students. The study identifies seven specific features of the HTP drawings that exhibit significant correlations with depressive symptoms in this population. As a result, the HTP test emerges as a potentially useful, easily applicable, and cost-effective tool for early screening of depression among middle school students.

Article [58] explores the significance of the “non-synthetic sign” in the S-HTP test as a possible indicator of neurodevelopmental disorders. Unlike the standard HTP test, the S-HTP requires participants to draw a house, a tree, and a human figure on the same page. The “non-synthetic sign” refers to an individual’s inability to integrate these three elements within a single drawing. The authors observed that difficulties or failures in completing the S-HTP test may indicate developmental impairments, with some correlation to lower IQ levels. Notably, the “non-synthetic sign” was observed with greater frequency in individuals diagnosed with ASD. Based on these findings, the authors suggest that the S-HTP test could serve as a valuable screening tool in educational settings, facilitating the early identification of developmental difficulties and enabling timely interventions for children at risk.

### 4.6. The Bears Family Test

Studies [42,66] build upon the research conducted by Iandolo and colleagues, who in 2012 developed a new projective construction test known as The Bear Family. In this test, participants are instructed to create and narrate a story using anthropomorphic bear-shaped dolls and scenic materials. This methodology enables children to organize and narrate a story, thereby offering valuable insights into their cognitive organization, narrative coherence, and predominant emotional and relational themes.

The test has been administered to typically developing children, children with ASD, and those with emotional difficulties. The findings from these studies indicate that the narratives produced during the projective test reflect the children’s emotional and relational experiences. These narratives can be used to identify difficulties, plan therapeutic interventions, and monitor progress over time. Furthermore, both studies propose that narrative therapy may serve as an effective approach to support children facing emotional and developmental challenges (e.g., those diagnosed with ASD or emotional difficulties), as it fosters emotional externalization, self-regulation skills, and problem-solving abilities.

Study [42] specifically examined the administration of this test to children and adolescents with ASD, comparing their performance with that of a TD control group. The results demonstrated that the narratives of children with ASD reflect their emotional states and personal experiences, particularly anxiety, and that their stories tend to emphasize maladaptive problems and behaviors more frequently than those of children with typical development. In summary, The Bear Family projective test appears to offer a meaningful perspective into the inner world of children with emotional difficulties, allowing for the identification of specific challenges in emotional processing and the development of targeted interventions aimed at improving their psychological well-being.

In article [66], the test was administered to children exhibiting emotional and relational difficulties, categorized as either inhibited or impulsive. The results indicated that children with emotional difficulties tended to construct narratives characterized by unresolved conflicts, unclear characters, negative relationships, and maladaptive behaviors. Unlike children without such difficulties, their stories lacked compensatory positive elements to counterbalance the negative themes. The study further revealed that the nature of emotional difficulties (inhibition vs. impulsivity) influenced the content of the stories, revealing distinct narrative patterns associated with each type of emotional challenge.

Overall, the findings suggest that the narratives of children with ASD can provide important insights into their emotional and behavioral experiences, facilitating the identification of children at risk of anxiety or other psychological difficulties.

### 4.7. Drawn Story Technique

In study [53], the Drawn Story Technique is presented as a projective test, administered alongside another technique known as Class Drawing, to primary and secondary school children. The findings indicate that these projective techniques serve as valuable tools for assessing potential risk factors—both at the individual and collective levels—that may contribute to psychological distress. Furthermore, these methods may prove beneficial in evaluating students’ emotional states and their adaptation to the school environment. The study further demonstrates that, despite certain limitations, these projective drawing techniques can contribute to enhancing children’s emotional well-being, academic adjustment, and overall school integration. By providing meaningful insights that foster dialogue with children, these methods help facilitate greater self-awareness of emotional states while fostering an environment of trust and cooperation within the school setting.

### 4.8. The Bird’s Nest Drawing

In study [75], the use of the projective test BND was examined in samples with diverse developmental backgrounds. The BND was shown to be a valuable tool for analyzing the emotional and cognitive world of children and adolescents. Findings indicated that the way children depict a bird’s nest reflects significant differences in their emotional state and relational experiences. For instance, children with psychiatric disorders struggled more in portraying the nest as a protective and reassuring space, whereas those with typical development created more detailed and secure drawings. Additionally, use of color, self-assessment of the drawing, and expert interpretation of emotional expression highlighted further differences among the groups. Overall, the BND emerged as an effective projective test for exploring children’s inner world, providing insights into attachment, emotional security, and relational perception. However, the study also highlighted some limitations, such as the small sample size and the need for more refined assessment criteria, underscoring the need for further research.

### 4.9. General Discussion

Although projective tests have declined in popularity in child and adolescent psychiatric diagnostics, and their use remains controversial, they continue to provide privileged access to emotional and conflictual issues that may be difficult to detect through interviews or standardized questionnaires. These tests allow for the assessment of unconscious psychological processes, facilitating the identification of vulnerabilities, psychological resources, and individual functioning patterns, thereby offering a comprehensive representation of underlying psychological dynamics. Projective techniques enable access to latent psychological content through the analysis of manifest responses and narratives provided by the patient. Additionally, they contribute to understanding an individual’s representation of their inner world, motivations, emotional experiences, and cognitive processes.

Projective tests can also provide valuable insights into interpersonal relationships and the presence of internal conflicts. Furthermore, they serve as complementary tools that enrich data obtained from standardized questionnaires and checklists, enabling a more comprehensive assessment of the individual. In clinical settings, these tests can be particularly useful for identifying relevant emotional themes and specific psychological dynamics. The use of images or narrative-based stimuli acts as a mediator, facilitating the expression of emotions and thoughts that may be challenging to articulate verbally. Among children and adolescents, projective techniques can reveal aspects of personality and conflicts related to key developmental stages. Some authors also suggest that projective tests can be employed as therapeutic tools, providing patients with a space for self-reflection and emotional processing. Moreover, certain scholars propose that not only physicians but health professionals in general may use these instruments for screening purposes in complex psychosocial situations.

Some authors emphasize that drawing-based projective tests offer greater versatility in developmental-age assessments for several reasons:

- Shorter administration times;
- Suitability for group contexts;
- Ability to overcome language barriers or verbal expression difficulties;
- Greater engagement and appeal for children, and improved acceptability among adolescents;
- Cost-effectiveness.

Nonetheless, many researchers highlight important considerations and limitations in the application of projective techniques. Over-interpretation should be avoided, as the narratives elicited by these tests are influenced by multiple factors, including the content of stimuli used in the test, the testing situation, and the individual’s internal world. Accurate interpretation requires specialized training and in-depth knowledge of projective methodologies; the mechanical application of predefined interpretation “recipes” is inadequate. It is crucial to integrate projective test results with additional information, such as anamnestic data, clinical observations, and other psychological assessments. Projective data should not be relied upon in isolation but must be cross-referenced with more objective psychometric measures. Additionally, cultural context plays a significant role in shaping test responses, and the associations and stories generated by children must be analyzed within their specific cultural context.

One of the primary limitations of projective tests is their lack of standardized psychometric criteria. These techniques are often criticized for their limited validity and reliability compared to standardized assessments, as they frequently fail to meet the criteria necessary for objective diagnostic classification. Another concern relates to interpretative bias, as results may be influenced by the subjectivity of the examiner, potentially leading to inconsistent outcomes. Furthermore, there is a risk of overgeneralization; while general trends may be observed, findings must always be interpreted in consideration of the specificity of each individual case. Lastly, projective techniques pose challenges in standardization and empirical validation, restricting their application in research contexts.

Although we employed an adapted Newcastle-Ottawa Scale (NOS) to assess study quality and risk of bias, we recognize that this approach may not fully capture all dimensions of bias; therefore, future systematic reviews should incorporate additional formal bias assessment tools. Additionally, conducting meta-analyses on specific aspects of construction projective techniques, such as inter-rater reliability and validity, could provide a more robust quantitative evaluation of their psychometric properties and clinical applicability.

## 5. Conclusions

In conclusion, our review suggests that projective construction techniques can provide valuable insights into the inner worlds of children and adolescents when used as complementary tools alongside validated psychometric assessments. However, the evidence supporting their utility is preliminary. Many of these historically established instruments have not undergone a significant re-evaluation of their psychometric properties since 2010, and nearly half of the studies analyzed did not offer detailed psychometric data. As a result, these tests should not be regarded as standalone diagnostic instruments, but rather as adjuncts that may enhance clinical and screening practices when applied by trained professionals. Their cost-effectiveness and non-invasive nature have led to widespread use in diverse contexts—from school and community settings to various clinical populations. Nonetheless, it is important to acknowledge both the limitations of the tests themselves and those inherent to the current body of evidence. Future research should aim to provide more robust empirical support and comprehensive evaluations of the psychometric properties of these tools. In clinical contexts, however, projective tests may be effectively combined with validated psychometric assessments to provide a deeper and more comprehensive understanding of the patient. When correctly administered by trained professionals, projective techniques can serve as windows into the unconscious world of children and adolescents, fostering an environment for listening, self-reflection, and psychological support. This safe space for emotional expression, increasingly difficult to achieve in contemporary society, could now be more essential than ever, as the demand for mental health support continues to rise among younger populations.

## Figures and Tables

**Table 1:** Overview of projective techniques categorized by the five major subtypes [1]. Two Examples of each subtype are provided.

Category	Category Description	Example	Test Description
Construction	They require individuals to create a narrative or an image based on a visual stimulus. They help explore thought processes, relationship perceptions, and internal conflicts.	Draw a Person Test (Machover, 1949) [9]	Participants are asked to draw a person on a blank sheet and then another person of the opposite sex.
		Thematic Apperception Test (Murray, 1938) [4]	Various ambiguous social scenes are shown, and participants are asked to create a story about the characters.
Association	They present an ambiguous stimulus and ask individuals to associate it with the first thing that comes to mind. The interpretation is based on the content and the cognitive processes activated by the individual.	Rorschach Inkblot Test (Rorschach, 1921) [10]	Participants are shown 10 symmetrical inkblots (5 black and white, 5 colored) and are asked what they resemble.
		Hand Test (Wagner, 1962) [11]	Various drawings of hands in different positions are shown, and participants are asked what they think they are doing.
Completion	They provide incomplete stimuli that the individual must complete, revealing unconscious themes and emotional dynamics. They analyze the structure of thought and the symbolic content of responses.	Washington University Sentence Completion Test (Loevinger, 1976) [12]	Participants are presented with incomplete sentences and asked to complete them.
		Rosenzweig Picture Frustration Study (Rosenzweig, Fleming, Clark, 1934) [13]	Cards depicting various frustrating situations are shown, and participants are asked how they would respond verbally.
Selection	The individual is required to select or arrange elements based on personal preferences or judgments. They examine emotional, motivational, and cognitive aspects.	Szondi Test (Szondi, 1935) [14]	Photos of psychiatric patients are shown, and participants are asked which one they like most and least.
		Luscher Color Test (Luscher, Scott, 1969) [15]	Participants are asked to rank a set of colored cards according to their preference.
Expression	They allow individuals to express themselves freely through drawing, play, or writing. They are useful for assessing the internal world and affective relationships in a non-verbal manner.	Projective Puppet Play (Woltmann, 1960) [16]	Children are asked to play different roles, such as parents or themselves, using puppets.
		Handwriting Analysis (Beyerstein, Beyerstein, 1992) [17]	Participants are asked to write spontaneous sentences to analyze their handwriting.

**Table 2:** Overview of the construction projective techniques identified in this systematic review. Each test is briefly described, highlighting its purpose, target population, and key interpretative aspects. References indicate the primary sources associated with each technique.

Test	Description	Ref
Thematic Apperception Test (TAT)	Developed by Murray and Morgan, the TAT consists of 20 picture cards depicting ambiguous and emotionally charged scenes. Participants are asked to create a story for each card, detailing what led up to the scene, what is occurring, and what might happen next. This narrative approach is designed to uncover underlying emotions, conflicts, and drives, though interpretative methods vary among practitioners.	[25,26,27,28,29]
Children’s Apperception Test (CAT)	A child-specific variant of the TAT, the CAT was introduced by Leopold and Sonia Bellak in 1965. It features 10 standardized cards that present scenarios tailored to children aged 7–12. Through the stories they construct, children reveal insights into their early psychosexual conflicts, emotional state, and coping strategies, offering a window into their interpersonal and intrapersonal dynamics.	[30,31,32]
Draw a Man Test (DAM)	Originally developed by Florence Goodenough in 1926, the DAM requires children to draw a complete human figure. The test is used to assess cognitive development by examining the accuracy, detail, and proportionality of the drawing’s features, which are believed to correlate with the child’s developmental stage.	[24]
Draw a Person Test (DAP)	Adapted by Karen Machover in 1949 from the DAM, the DAP shifts the focus toward personality assessment. In this test, children draw a person, and the resulting image is interpreted as a projection of their self-image and emotional dynamics. The analysis looks for indications of underlying emotional difficulties, self-esteem issues, interpersonal perceptions, and trauma related to attachment figures. It can be interpreted using two main approaches: Machover’s analytical method, which links drawing details to personality traits and psychopathology, and Koppitz’s global approach, which evaluates several indicators to generate an overall score.	[9,33,34,35,36,37,38]
Draw a Family Test (DAF)	Developed by Miles Porot in 1951, the DAF asks children (5 to 16 years of age) to draw their family. The test aims to assess the child’s perceptions of familial roles, dynamics, and communication patterns. Analysis of the drawing can reveal insights into the child’s emotional state, attachment styles, and potential areas of family conflict or support.	[39]
House Tree Person (HTP)	Originally created by John Buck in 1948 and later revised in collaboration with Emanuel Hammer in 1969, to revise and expand the test, enhancing the interpretation criteria to improve its applicability. The HTP involves drawing three separate images: a house, a tree, and a person. Each element is symbolically analyzed—the house as a representation of self and security, the tree as an indicator of growth and stability, and the person as a reflection of social interactions and self-concept. Through drawing, individuals can recreate and externalize their emotional experiences.	[40,41]
The Bears Family Projective Test	The most recent method reviewed, this test is designed for children aged 3–11. It utilizes a set of bear characters and distinct living environments to stimulate play and storytelling. The administration includes two five-minute play sessions followed by a narrative session, during which the child is encouraged to tell a story based on their play. It provides an assessment of children’s emotional world and family relationships.	[42,43,44,45]
Drawn Story Technique	Introduced by Trombini in 1994, this technique prompts children and adolescents to create a sequence of drawings that together form a story. The evaluation of the resulting narratives is based on clearly defined criteria, such as the the content and structure of the story, and its conclusion, which provide insights into the child’s inner emotional world and level of emotional distress.	[46,47]
Bird’s Nest Drawing (BND)	The BND is a single-drawing measure based on attachment theory and used to assess the cognitive, emotional, and behavioral aspects of internalized early childhood experiences. Children are asked to draw a bird’s nest, which serves as a metaphor for early relational experiences. The drawing is evaluated using Kaiser’s checklist, which quantifies both structural and thematic indicators that relate to perceptions of safety, nurturance, and interpersonal closeness.	[48,49,50,51]

**Table 3:** Detailed description of the studies included in the systematic review, with quality assessment (QA, see supplementary Tables S1 and S2 for further details).

Study	Year	Design	Sample Characteristics	Projective Techniques	Other Materials	Main Results	QA
[42]	2012	Case-control	40 Italian children (5–9 years) with emotional and relational difficulties and 322 TD children (4–10 years).	The Bears Family (3 × 5 min sessions)	WPPSI or WISC-R (intelligence), CBCL, TRF (emotional-behavioral problems)	Children with emotional difficulties showed more unresolved problems, unclear characters, and predominantly negative content in their stories. Inter-rater reliability was assessed in 25% of the sample and was considered acceptable (Cohen’s kappa = 0.96).	7/9
[52]	2023	Cross-sectional	167 Chinese students (ages 12–14)	HTP (freehand drawing associated with semi-structured interview)	CES-D; PRCPS	Eight drawing characteristics (e.g., lack of movement, lack of detail, darkening of the paper) appeared with significant higher frequency in the depressed group. Seven of these were significant independent predictors of depressive symptoms (Nagelkerke R^2^ 0.471, overall prediction rate 83.2%).	6/9
[53]	2023	Cross-sectional	1757 children from Sicilian schools: 1270 in primary school (6–10 years) and 487 in secondary school (11–13 years).	Drawn Story Technique and Classroom Drawing (children freely depict their class as preferred) (45 min total)	-	The Drawn Stories Technique revealed significant gender differences: males produced a higher proportion of Negative Outcomes compared to females, particularly in primary school settings. The Classroom Drawing provided limited insight into individual differences in scholastic integration overall. No further psychometric validation details are provided.	3/9
[54]	2023	Qualitative	60 immigrant children (ages 4–14) from Afghanistan, Syria, and Iraq living in Turkey.	DAF (no time limit)	Family information form and face-to-face interviews	Three main themes (Chaos, Necessity, Development) and nine sub-themes were identified (e.g., Interpersonal Relations, Thoughts about Future, Violence, Emotional State). Examining DAF tests, several family relations of immigrant children were considered negatively affected.	2/9
[55]	2018	Qualitative	15 children aged between 5 and 10 years	DAFPT: drawings were analyzed based on sequence and gender factors (DAP), maturity level (DAM), and collocation and rotation factors (Bender-Gestalt Test).	-	Psychometric evaluation of this novel tool based on expert and parental fitness ratings using a 5-point scale, where higher scores indicate a better fit. Both experts and parents rated the test as “very fit” for assessing various dimensions of children’s attitudes toward their family (overall weighted means above 4.2).	2/9
[56]	2014	Case-control	80 children (40 with ADHD and 40 without), aged 9–10 years, attending third and fourth grade in schools in Shiraz, Iran.	DAP and DAF (with drawing times recorded)	DSM-IV ADHD questionnaire for teachers and parents	Children with ADHD showed higher impulsivity and emotional issues in their drawing tests in comparison with TD children. No new psychometric data on the DAP were reported.	6/9
[57]	2024	Cross-sectional	25 individuals with PWS, with 13 participants between 12 and 18 years old.	HTP (house, tree, person in 3 different pictures) and DAF (no time limit)	-	The house technique indicated emotional dysregulation, social withdrawal, oppositional tendencies, and anxiety in individuals with PWS. The tree technique revealed low self-esteem, impulsivity, relationship difficulties, frustration, and demotivation. No psychometric data reported.	3/9
[58]	2016	Retrospective	283 children (4–17 years) referred to a psychosomatic clinic	S-HTP (house, tree, person in one picture) or HTP (if unable to complete S-HTP)	WISC-III/IV (intelligence)	S-HTP group significantly differed from HTP group in intelligence scores and rates of autism diagnosis. Therefore the inability to complete S-HTP (“no-synthetic sign”) may indicate an underlying developmental disorder, such as autism spectrum disorder or a lower mental age. No further psychometric details.	4/9
[59]	2020	Cross-sectional	60 adolescents (aged 12–18) whose bullying had ceased for at least 1 month	Rorschach and TAT	Clinical interviews; FAD; CAPS-CA-5; SCL-90	A mixed methods approach with projective tests is presented as a promising method for understanding the complex psychological bullying experiences of adolescent victims. No data analysis available.	5/9
[60]	2018	Cross-sectional	30 children (ages 7–13) diagnosed with epilepsy for at least 3 months	DAP (2 drawings: one of themselves “today” and one from “before the illness”)	STAI-C (anxiety)	The mean STAI-C scores showed moderate anxiety (M = 14.3 SD = 4.21; range 0–22), which was confirmed by qualitative analysis of DAP. No further quantitative data about the DAP are provided.	3/9
[61]	2019	Longitudinal	281 very preterm infants (7–14 years) from the PREMAG study (prenatal magnesium sulfate’s effect)	DAM (51-items rating scale)	SDQ, Neuropsychomotor development questionnaire	The DAM results were similar in the magnesium sulfate group and in the placebo group. Lower DAM scores were associated with abnormal overall SDQ scores (sensibility 43%, specificity 72%, PPV 35%, NPV 75%) and with cognitive deficits (sensitivity 42%, specificity 82%, PPV 78%, NPV 48%).	5/9
[62]	2012	Case-control	72 very preterm and 60 term children, aged 5 years	DAP	WISC—RN—(Revised Netherlands)	The DAP demonstrated good psychometric properties (test-retest reliability 0.74; strong interrater reliability). It showed a moderate correlation (r = 0.40) with total IQ on the WISC-RN, indicating its potential as a screening tool for cognitive and motor development in young children	7/9
[63]	2023	Cross-sectional	34 Spanish children (5–11 years) receiving care from Specialized Services for risk of neglect or maltreatment.	DAP—T2F (2 drawings: a human figure and then one of the opposite sexes, with no time limit; with 52 developmental and 35 emotional indicators)	Raven intelligence test	T2F Developmental Indicators significantly correlated with Raven’s intelligence scores (*p* < 0.05; Cronbach’s coefficient 0.86, Spearman-Brown 0.86). Graphic indicators of child sexual abuse were found in most of the drawings, with Indicator 1 (Body omitted/distorted) being the most frequent (males > females). 53% of the sample had a high number of Emotional Indicators (>75 percentile), showing high risk of emotional problems.	4/9
[64]	2023	Cross-sectional	80 children (6–8 years) from a public school in Japan	DAP—T2F	Depression Self-Rating Scale for children	The DAM test scores showed significant differences in hyperadaptation levels among girls (*p* = 0.013) but not among boys. Specific graphic features, including mouth/nose/ears (*p* = 0.005), hair (*p* = 0.007), and fingers (*p* = 0.017), were more detailed in hyperadapted girls. The DAM test could be useful for identifying over-adaptation tendencies in girls aged 6–8 years. No further statistical data are available.	3/9
[65]	2020	Case-control	21 TD children and 22 children with ASD	DAM (3 drawings: man, a woman, and a self-portrait)	NEPSY-II, Affect recognition tasks	The DAM Maturity Scale scores were significantly lower in children with ASD compared to typically developing (TD) children (*p* < 0.001, η^2^ = 0.22–0.38). A significant correlation emerged between self-drawing maturity and affect recognition in ASD children (*p* < 0.05). The test discriminated between ASD and TD groups (*p* = 0.02), highlighting repetitive drawing patterns in children with ASD. No further psychometric validity data are available.	8/9
[66]	2020	Cross-sectional	50 Spanish children (5–18 years): 25 with ASD with adequate verbal skills, and 25 TD	The Bears Family Projective Test (bear dolls and props to create a story in 5 min; session recorded)	WISC-IV; CBCL; Reynolds RIAS intelligence test; SENA (Children and Adolescents Assessment System)	The analysis of narrative content showed that children with ASD reported more adaptive and maladaptive behaviors (*p* = 0.05), more episodes (*t* = −3.28, *p* = 0.02), and fewer external environmental elements in their stories compared to TD children. Narrative complexity correlated with chronological age in ASD children (*r* = 0.50, *p* = 0.01). Inter-rater reliability was high (Cohen’s κ = 0.91). The test demonstrated partial construct validity, correlating with anxiety symptoms.	5/9
[67]	2017	Cross-sectional	33 Turkish adolescents (14–17 years) admitted to University Hospital	TAT (four cards with seven questions each)	AAP, RSQ (attachment)	AAP scores showed high inter-rater reliability (*ICC* = 0.78–0.87) and classification stability (Cohen’s κ = 0.73, *p* < 0.001). Principal Component Analysis confirmed a single-factor structure explaining 63.09% of the variance. Significant correlations were found with four key attachment dimensions: Self-Other (*r* 0.74), Avoidance-Self (*r* 0.48), Avoidance-Other (*r* 0.59), and Self-Dependency (*r* 0.43). The test also showed moderate agreement (58%) with the RSQ. However, no reliability data were reported, and the limited comparison with standardized clinical tools restricts its psychometric validation.	3/9
[68]	2016	Cross-sectional	62 kindergarten students (5–7 years; 45 White, 4 African-American, 6 Hispanic, and 6 Asian	TAT (six pictures, coded with Teglasi’s system as a storytelling performance measure of executive functions)	WPPSI–III; NEPSY/NEPSY-II (executive functions); SSIS (social and academic skills), and BRI (teacher-rated executive function assessment)	The TAT assessed executive functions by simulating real-life judgment and response processes, making it a valuable tool for evaluating them in everyday contexts. TAT showed excellent interrater reliability (interclass correlation between 0.89 and 0.94). Internal consistency across the six cards was acceptable (range 0.76–0.91). The composite TAT score correlated with WPPSI Vocabulary (r 0.36), and with several NEPSY scales.	4/9
[69]	2022	Case-report	11-year-old adolescent female presenting with NSSI behaviors.	CAT-H (CAT with Human Figures; administered during the second session)	Seven semi-structured Winnicottian psychoanalytic interview sessions	The CAT-H facilitated interventive psychodiagnosis, helping the adolescent explore emotions. It may be effective for addressing self-harm in adolescents. No psychometric data or statistical analysis are reported.	2/9
[70]	2018	Cross-sectional	4 children (7–10 years) with ADHD under medical monitoring.	CAT-A (CAT with Animal figures; administered in 2 sessions)	-	CAT-A revealed family conflicts and relational difficulties, highlighting challenges in play and symbolization essential for emotional development. No psychometric data or statistical analysis are reported.	3/9
[71]	2023	Cross-sectional	99 children (5–12 years): 45 from a psychiatric inpatient unit and 54 from a public elementary school.	DAF and CAT (analyzed using the coding system for mental state talk—CS-MST)	ASCT (attachment)	The CAT assessed using the CS-MST demonstrated excellent reliability (ICC = 0.97), and the drawing assessment scales showed strong consistency (ICC = 0.95–0.97). It correlated with attachment classification in hospitalized children (r 0.36, *p* 0.02) and non-hospitalized children (r = 0.33, *p* = 0.02). DAF scores correlated with CAT—CS-MST in the hospitalized group (Content Scale: r = 0.31, *p* = 0.04; Formal Elements Scale: r = 0.40, *p* = 0.008). There was a marginally significant positive correlation between attachment quality and the Content scores of the family drawings (r 0.29, *p* 0.061). These findings suggest that the test can be useful for exploring attachment quality and mentalization development in children, particularly in clinical settings.	6/9
[72]	2014	Cross-sectional	The study reviewed medical records of 87 pediatric psychiatric patients with NSSI, analyzing their object relations retrospectively	TAT (cards 1–5; analyzed using the Social Cognition and Object Relations Scale-Global (SCORS-G) rating method)	The Hospitalized Child and Adolescent Trauma and Psychopathology Questionnaire (HCATP)	SCORS-G ratings of TAT responses showed high inter-rater reliability (Cronbach’s α > 0.85, ICC > 0.85). Adolescents with NSSI scored significantly higher in complexity of representations of people (F = 4.34, *p* < 0.05), and had greater understanding of social causality (F = 3.23).	4/9
[73]	2013	Longitudinal	30 patients (11–17 years) hospitalized in Paris after a suicide attempt.	Rorschach Test and TAT	Clinical interviews	The responses to projective tests showed significant changes over one year in adolescents after a suicide attempt (*p* = 0.0019, *p* = 0.0067). No psychometric data available for the TAT.	2/9
[74]	2015	Case-report	1 female patient, 5 years old, hospitalized for leukemia.	DAP:SPED	CDI (depression); MASC (anxiety)	The DAP:SPED has proved to be a valid tool for detecting emotional distress in children, with high sensitivity (84%) and specificity (89%), standing out for its ability to identify emotional problems that do not emerge in self-report tests. Its graphic indicators were selected based on their rarity in neurotypical children (<5%, *p* < 0.05) and show a strong correlation with clinical diagnoses (*r* = 0.68, *p* < 0.01). Additionally, the test significantly discriminated between children with and without emotional disorders (*t* = 5.21, *p* < 0.001) and ensured reliable assessment among examiners (ICC > 0.85).	5/9
[75]	2024	Case-control	18 patients with schizophrenia (11–16 y); 45 outpatients with behavioral issues (7–16 y); 8 TD children conceived with assisted reproductive technologies (5–13 y); 14 TD controls.	BND	-	BND parameters showed significant differences between groups (*p* ≤ 0.05; φ criterion). Differences were found in the use of colors, self-assessment of the drawing, and the evaluation of emotional expression by experts. Children in the control group used more colors, rated their drawings more positively, and received better emotional evaluations from experts, indicating greater emotional expressiveness. In contrast, hospitalized patients showed limited use of colors, a negative perception of their drawings, and lower emotional expression. Children conceived through assisted reproductive technologies and outpatient clients obtained intermediate results.	7/9

Abbreviations: AAP, Adolescent Attachment Protocol; ADHD, Attention-Deficit/Hyperactivity Disorder; ASCT, Attachment Story Completion Task; ASD, Autism Spectrum Disorder; BRI, Behavior Rating Inventory; BRIEF, Behavior Rating Inventory of Executive Function; CAPS-CA-5, Clinician-Administered PTSD Scale for DSM-5—Child/Adolescent Version; CAT, Children’s Apperception Test; CBCL, Child Behavior Checklist; CDI, Children’s Depression Inventory; CES-D, Center for Epidemiologic Studies Depression Scale; CSI-4, Child Symptom Inventory-4; DAF, Draw a Family test; DAFPT, Draw-a-Family Picture Test; DAM, Draw a Man test; DAP, Draw a Person test; FAD, Family Assessment Device; HTP, House Tree Person Drawing; MASC, Multidimensional Anxiety Scale for Children; NPV, Negative Predictive Value; NSSI, Non-suicidal self-injury; PPV, Positive Predictive Value; PRCPS, Perceived Risk of COVID-19 Pandemic Scale; PWS, Prader-Willi Syndrome; RSQ, Relationship Scales Questionnaire; SCL-90, Symptom Checklist-90-Revised; SDQ, Strengths and Difficulties Questionnaire; STAI-C, State-Trait Anxiety Inventory for Children; T2F, Two Human Figures test; TD, typically developing; TRF, Teacher’s Report Form; WISC-R, Wechsler Intelligence Scale for Children—Revised; WPPSI, Wechsler Preschool and Primary Scale of Intelligence.

## Data Availability

The original contributions presented in this study are included in the article/Supplementary Material. The raw data supporting the conclusions of this article will be made available by the authors on request.

## Supplementary Materials

The following supporting information can be downloaded at: https://www.mdpi.com/article/10.3390/children12040406/s1, Table S1: Adapted version of the Newcastle-Ottawa scale used in the present study for quality assessment (maximum 9 stars); Table S2: Details on quality assessment indices for the retrieved studies.

## Abbreviations

The following abbreviations are used in this manuscript:

AAP	Adolescent Attachment Protocol
ADHD	Attention-Deficit/Hyperactivity Disorder
ALL	Acute Lymphoblastic Leukemia
ASD	Autism Spectrum Disorder
BND	Bird’s Nest Drawing
BRI	Behavior Rating Inventory
BRIEF	Behavior Rating Inventory of Executive Function
CAPS-CA5	Clinician-Administered PTSD Scale for DSM-5—Child/Adolescent
CAT	Child Apperception Test
CAT-A	Child Apperception Test—Animal form
CAT-H	Child Apperception Test—Human figure form
CBCL	Child Behavior Checklist
CDI	Children’s Depression Inventory
CES-D	Center for Epidemiologic Studies Depression Scale
CSI-4	Child Symptom Inventory-4
DAM	Draw a Man
DAF	Draw a Family
DAFPT	Draw a Family Picture Test
DAP	Draw a Person
DAP:SPED	Draw a Person: Screening Procedure for Emotional Disturbance
DSM	Diagnostic and Statistical Manual of Mental Disorders
EF	Executive Functions
FAD	Family Assessment Device
HTP	House Tree Person
IQ	Intelligence Quotient
MASC	Multidimensional Anxiety Scale for Children
MDPI	Multidisciplinary Digital Publishing Institute
NNSI	Non-Suicidal Self-Injury
PEP	Psychoanalytic Electronic Publishing
PRCPS	Perceived Risk of COVID-19 Pandemic Scale
PTSD	Post-Traumatic Stress Disorder
PWS	Prader-Willi Syndrome
RSQ	Relationship Scales Questionnaire
S-HTP	Synthetic- House Tree Person
SCL-90	Symptom Checklist-90-Revised
SSIS	Social Skills Improvement System
STAI-C	State-Trait Anxiety Inventory for Children
T2F	Two Human Figures Test
TD	Typically Developing
TAT	Thematic Apperception Test
TD	Typical Development
TRF	Teacher’s Report Form
WISC-R	Wechsler Intelligence Scale for Children—Revised
WPPSI	Wechsler Preschool and Primary Scale of Intelligence
