# Supplementary material for: Projective in Time: A Systematic Review on the Use of Construction Projective Techniques in the Digital Era—Beyond Inkblots
Source: Children (Basel). 2025 Mar 24;12(4):406. doi: 10.3390/children12040406 (PMC12025577; doi:10.3390/children12040406)
Supplement: Supplementary file 1 [file children-12-00406-s001.zip › children-3483178-supplementary.docx]

Review

Projective in Time: A Systematic Review on the Use of
Construction Projective Techniques in the Digital
Era—Beyond Inkblots

| Academic Editors: Benedetto Vitiello  Received: 01 February 2025  Revised: 21 March 2025  Accepted: 22 March 2025  Published: 24 March 2025  **Citation:** Santillo, G.; Morra, R.C.; Esposito, D.; Romani, M. Projective in Time: A Systematic Review on the Use of Construction Projective  Techniques in the Digital Era —Beyond Inkblots. *Children* **2025**, *12*, x. https://doi.org/10.3390/xxxxx  **Copyright:** © 2025 by the authors. Submitted for possible open access publication under the terms and conditions of the Creative Commons Attribution (CC BY) license (https://creativecommons.org/licenses/by/4.0/). |
| --- |

Giada Santillo, Rita Chiara Morra, Dario Esposito * and Maria Romani

Department of Human Neuroscience, Sapienza University of Rome, 00185 Rome, Italy;
giada.santillo@uniroma1.it (G.S.); ritachiara.morra@uniroma1.it (R.C.M.); maria.romani@uniroma1.it (M.R.)

***** Correspondence: dario.esposito@uniroma1.it

**Supplementary Material**

**Table 1S**

Adapted version of the Newcastle-Ottawa scale used in the present study for quality assessment (maximum 9 stars).

| **NEWCASTLE - OTTAWA QUALITY ASSESSMENT SCALE**  **Selection** (max 4)  1) Is the case definition adequate?  a) yes, with standard diagnostic criteria (i.e., DSM, ICD, published diagnostic criteria) or valid assessment (both for patients and healthy subjects). **✵**  b) yes, based on caregivers/self-reports or on no valid clinical assessment.  d) no description.  2) Representativeness of the cases  a) consecutive or obviously representative series of cases. **✵**  b) potential for selection biases or not stated.  3) Selection of Controls/Other groups  a) community controls **✵**  b) hospital controls  c) no description or absence of controls or other experimental groups  4) Definition of Controls (max 1)  a) no history of disease (healthy controls) **✵**  b) other diagnoses  c) no description of source or absence of controls or other experimental groups  **Comparability** (max 2)  1) Comparability of the sample on the basis of the design or analysis  a) study controls for age and sex **✵**  b) study controls for other psychological dimensions, assessed with non-projective standardized tests **✵**  c) no description  **Methods and procedure**   1. Experimental procedure:   a) The experimental procedure is well described and includes valid measures and methods. **✵**  b) Poor description or no description of the procedure.  2) Same procedure for cases and controls:  a) yes **✵**  b) no  c) No description or absence of controls  3) Non-respondent rate   1. Non-respondents described with reasons – if a control group is present, non-respondent rate is the same for both groups. **✵**   b) rate different and/or no designation/explanation. |
| --- |

**Table 2S**

Details on quality assessment indices for the retrieved studies.

| **Study** | **Selection (max 4*)** | | | | **Comparability (max 2*)** | **Methods and procedure (max 3*)** | | | **Total (max 9*)** |
| --- | --- | --- | --- | --- | --- | --- | --- | --- | --- |
|  | **Is the case definition adequate?** | **Representativeness of the cases:** | **Selection of controls/** **Other groups:** | **Definition of controls:** | **Comparability of cases and controls on the basis of the design or analysis** | **Experimental procedure:** | **Same experimental procedure for cases and controls:** | **Non-respondent rate** |  |
| Shin, H. and Lehmkuhl, G. (2021) | (b) | (b) | (c) | (c) | (c) | (a) | (a) | (b) | 2 |
| Iandolo, G. et al. (2012) | *(a) | (b) | *(a) | *(a) | **(a,b) | *(a) | *(a) | (b) | 7 |
| Guo, Q. et al. (2023) | *(a) | *(a) | (b) | *(a) | *(a) | *(a) | *(a) | (b) | 6 |
| La Gutta, S. et al. (2023) | (b) | (b) | (c) | (b) | *(a) | *(a) | *(a) | (b) | 3 |
| Efe, Y.S. et al. (2023) | (b) | (b) | (c) | (c) | (c) | *(a) | (c) | *(a) | 2 |
| Policarpio-Gutierrez, M. (2018) | (b) | (b) | (c) | (c) | (c) | *(a) | (c) | *(a) | 2 |
| Haghighi, M. et al. (2014) | *(a) | (b) | *(a) | *(a) | *(a) | *(a) | *(a) | (b) | 6 |
| Gonzalez-Ruiz, Y. et al. (2024) | *(a) | (b) | (c) | (c) | (c) | *(a) | (c) | *(a) | 3 |
| Fujii, C. et al. (2016) | *(a) | (b) | (c) | (b) | *(a) | *(a) | *(a) | (b) | 4 |
| Roques, M. et al. (2020) | *(a) | (b) | (c) | (c) | **(a,b) | *(a) | *(a) | (b) | 5 |
| Hamama, L. and Alshech, M. (2018) | *(a) | *(a) | (c) | (c) | (c) | *(a) | (c) | (b) | 3 |
| Chollat, C. et al. (2019) | *(a) | *(a) | (b) | (b) | *(a) | *(a) | *(a) | (b) | 5 |
| Schepers, S. et al. (2012) | *(a) | (b) | *(a) | *(a) | **(a,b) | *(a) | *(a) | (b) | 7 |
| Ballús, E. et al. (2023) | *(a) | *(a) | (c) | (c) | *(a) | *(a) | (c) | (b) | 4 |
| Horiuchi, F. et al. (2023) | (b) | (b) | (c) | (c) | *(a) | *(a) | *(a) | (b) | 3 |
| Papangelo, P. et al. (2020) | *(a) | *(a) | *(a) | *(a) | **(a,b) | *(a) | *(a) | (b) | 8 |
| Iandolo, G. et al. (2020) | *(a) | (b) | *(a) | *(a) | *(a) | *(a) | (b) | (b) | 5 |
| Tüzün, Z. and Soygüt, G. (2017) | *(a) | *(a) | (c) | (c) | (c) | *(a) | (c) | (b) | 3 |
| Annotti, L.A. and Teglasi, H. (2016) | *(a) | *(a) | (c) | (c) | (c) | *(a) | *(a) | (b) | 4 |
| Chaves, G. et al. (2022) | * (a) | (b) | (c) | (c) | (c) | *(a) | (c) | (b) | 2 |
| Tonetto, A.P. et al. (2018) | *(a) | *(a) | (c) | (c) | (c) | *(a) | (c) | (b) | 3 |
| Rosen, H. et al. (2023) | *(a) | (b) | *(a) | *(a) | *(a) | *(a) | *(a) | (b) | 6 |
| Conway, F. et al. (2014) | *(a) | *(a) | (b) | (b) | (c) | *(a) | *(a) | (b) | 4 |
| De Kernier, N. (2013) | *(a) | *(a) | (c) | (c) | (c) | (b) | (c) | (b) | 2 |
| Herrmann, S. (2015) | *(a) | (b) | (c) | (c) | **(a,b) | *(a) | (c) | *(a) | 5 |
| Zvereva, M. et al. (2024) | *(a) | *(a) | *(a) | *(a) | *(a) | *(a) | *(a) | (b) | 7 |
| ***Note:* the specific fulfilled criterion for the assignment or non-assignment of each star is specified in brackets.** | | | | | | | | | |
